# Machine Learning in Medical Imaging

**DOI:** 10.1155/2012/123727

**Published:** 2012-03-05

**Authors:** Kenji Suzuki, Pingkun Yan, Fei Wang, Dinggang Shen

**Affiliations:** ^1^Department of Radiology, University of Chicago, Chicago, IL 60637, USA; ^2^Xian Institute of Optics and Precision Mechanics, Chinese Academy of Sciences, Shaanxi, Xi'an 710119, China; ^3^IBM Almaden Research Center, San Jose, CA 95120, USA; ^4^Department of Radiology and BRIC, University of North Carolina at Chapel Hill, Chapel Hill, NC 27599, USA

Medical imaging is becoming indispensable for patients' healthcare. Machine learning plays an essential role in the medical imaging field, including computer-aided diagnosis, image segmentation, image registration, image fusion, image-guided therapy, image annotation, and image database retrieval. With advances in medical imaging, new imaging modalities and methodologies such as cone-beam/multislice CT, 3D ultrasound imaging, tomosynthesis, diffusion-weighted magnetic resonance imaging (MRI), positron-emission tomography (PET)/CT, electrical impedance tomography, and diffuse optical tomography, new machine-learning algorithms/applications are demanded in the medical imaging field. Because of large variations and complexityit is generally difficult to derive analytic solutions or simple equations to represent objects such as lesions and anatomy in medical images. Therefore, tasks in medical imaging require “learning from examples” for accurate representation of data and prior knowledge. Because of its essential needs, machine learning in medical imaging is one of the most promising, growing fields.

The main aim of this special issue is to help advance the scientific research within the broad field of machine learning in medical imaging. The special issue was planned in conjunction with the International Workshop on Machine Learning in Medical Imaging (MLMI 2010) [1], which was the first workshop on this topic, held at the 13th International Conference on Medical Image Computing and Computer-Assisted Intervention (MICCAI 2010) in September, 2010, in Beijing, China. This special issue is one in a series of special issues of journals on this topic [2]; it focuses on major trends and challenges in this area, and it presents work aimed at identifying new cutting-edge techniques and their use in medical imaging.

The quality level of the submissions for this special issue was very high. A total of 17 papers were submitted to this issue in response to the call for papers. Based on a rigorous review process, 10 papers (59%) were accepted for publication in the special issue. The special issue starts by a review of studies on a class of machine-learning techniques, called pixel/voxel-based machine learning, in medical imaging by K. Suzuki. A series of medical imaging applications of machine-learning techniques are presented. A large variety of applications are well represented here, including organ modeling by D. Wang et al. and X. Qiao and Y.-W. Chen, brain function estimation by V. Michel et al., image reconstruction by H. Shouno et al., lesion classification by P. Wighton et al., modality classification by X.-H. Han and Y.-W. Chen, lesion segmentation by M. Zortea et al., organ segmentation by S. Alzubi et al., and visualization of molecular signals by F. Mattoli et al. Also, the issue covers various biomedical imaging modalities, including MRI by D. Wang et al., CT by X. Qiao and Y.-W. Chen and H. Shouno et al., functional MRI by V. Michel et al., dermoscopy by P. Wighton et al. and M. Zortea et al., scintigraphy by X.-H. Han and Y.-W. Chen, ultrasound imaging by X.-H. Han and Y.-W. Chen, radiography by X.-H. Han and Y.-W. Chen, MR angiography by S. Alzubi et al., and microscopy by F. Mattoli et al. as well as a variety of organs, including the kidneys by D. Wang et al., liver by X. Qiao and Y.-W. Chen, brain by V. Michel et al. and F. Mattoli et al., chest by S. Alzubi et al., skin by P. Wighton et al. and M. Zortea et al., and heart by F. Mattoli et al. Various machine-learning techniques were developed/used to solve the respective problems, including structured dictionary learning by D. Wang et al., generalized N-dimensional principal component analysis by X. Qiao et al., multiclass sparse Bayesian regression by V. Michel et al., Bayesian hyperparameter inference by H. Shouno et al., supervised learning of probabilistic models based on maximum aposteriori estimation and conditional random fields by P. Wighton et al., joint kernel equal contribution in support vector classification by X.-H. Han and Y.-W. Chen, and iterative hybrid classification by M. Zortea et al.

We are grateful to all authors for their excellent contributions to this special issue and to all reviewers for their reviews and constructive suggestions. We hope that this special issue will inspire further ideas for creative research, advance the field of machine learning in medical imaging, and facilitate the translation of the research from bench to bedside.

*Kenji Suzuki* *Kenji Suzuki* *Pingkun Yan* *Pingkun Yan* *Fei Wang* *Fei Wang* *Dinggang Shen* *Dinggang Shen*
